# Supplementary material for: Symbiotic polyamine metabolism regulates epithelial proliferation and macrophage differentiation in the colon
Source: Nat Commun. 2021 Apr 8;12:2105. doi: 10.1038/s41467-021-22212-1 (PMC8032791; doi:10.1038/s41467-021-22212-1)
Supplement: Supplementary file 1 — Supplementary Information [file 41467_2021_22212_MOESM1_ESM.pdf]

Supplementary Information

Symbiotic polyamine metabolism regulates epithelial proliferation and macrophage differentiation in the colon

Atsuo Nakamura et al.

- Supplementary Figures 1-9
- Supplementary Tables 1-2

Supplementary figure 1

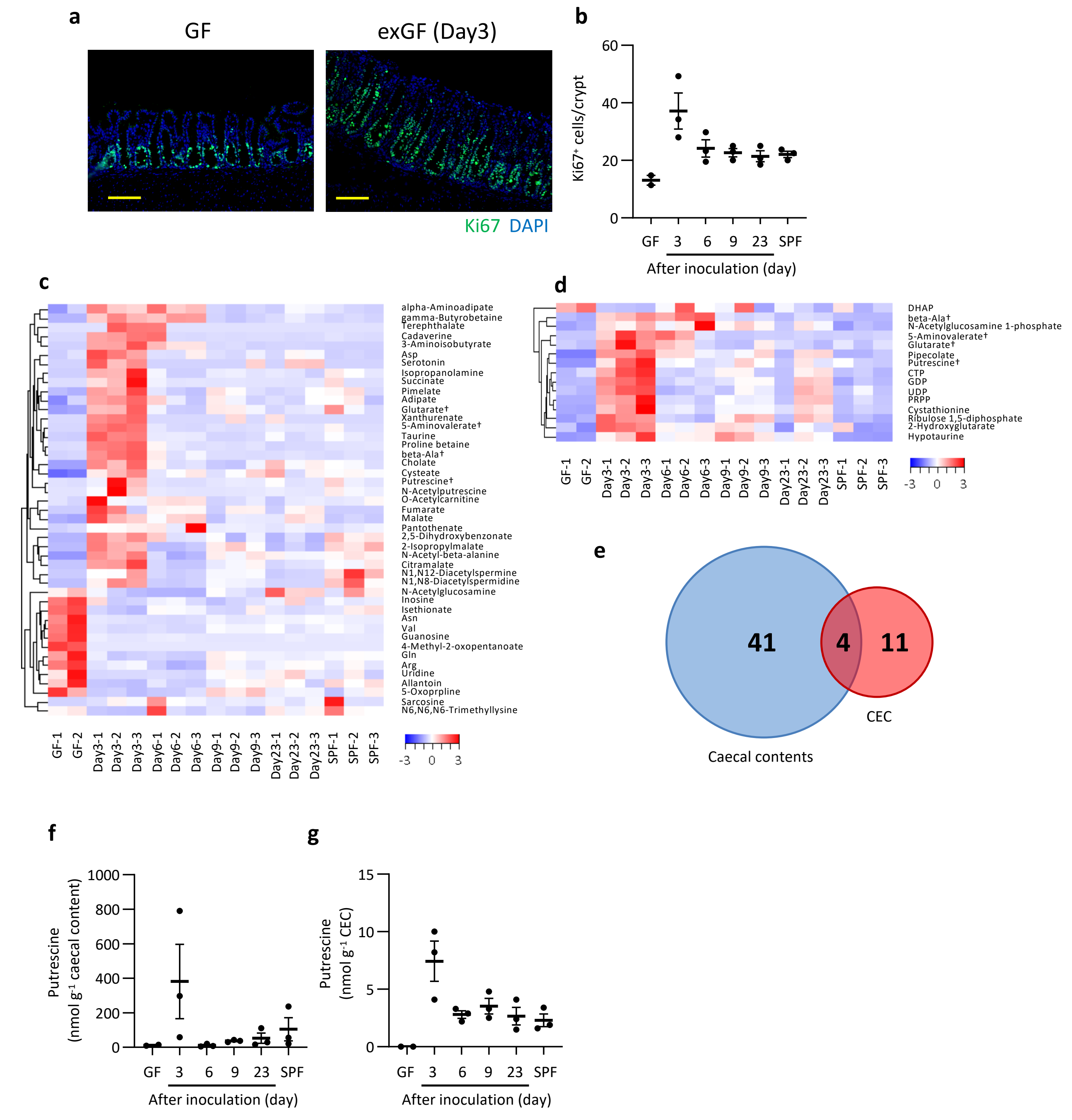

Supplementary Fig. 1. CECs proliferation correlated with intracellular and luminal putrescine.
a Representative microscopic images of Ki67 immunostaining (green) in colonic tissues of GF, exGF and SPF mice. Nuclei are counterstained with DAPI (blue). Scale bar: 100 µm. **b** Quantification of Ki67-positive cells/crypt per group (GF: n = 2, day 3–23 and SPF: n = 3, independent animals). **c, d** Heatmaps indicate the metabolites significantly correlated with the number of Ki67-positive cells in caecal contents (**c**) or colonic epithelial cells (**d**). Correlations were analyzed using the Spearman's rank correlation coefficient. **e** Venn diagram indicates the metabolites positively correlated with the number of Ki67-positive cells in cecal contents and CEC. †Metabolites commonly found in **c** and **d**. **f, g** Putrescine concentration in **f** caecal contents or **g** CECs of GF, exGF and SPF mice (GF: n = 2, day 3–23 and SPF: n = 3). Data shown represent the mean ± SEM.

Supplementary figure 2

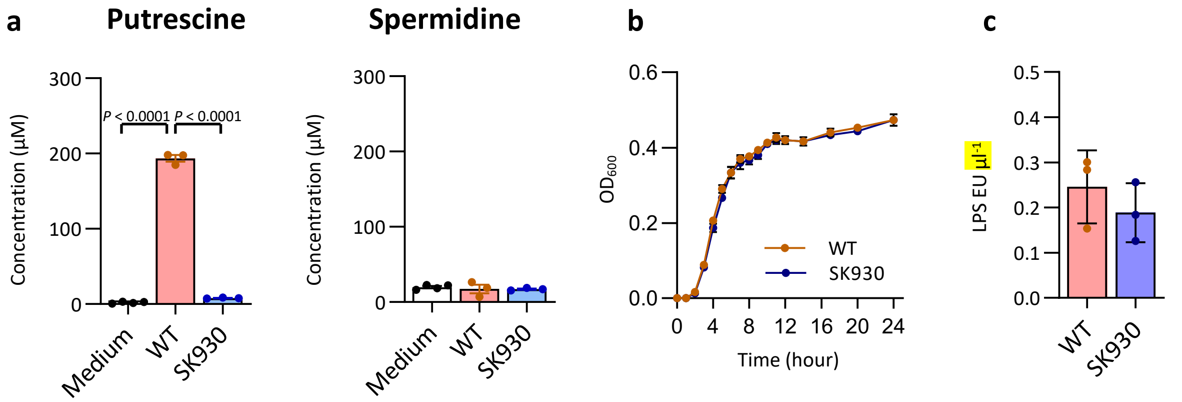

Supplementary Fig. 2. Characteristics *E. coli* WT and SK930 strain.

a The putrescine and spermidine concentrations in of WT and SK930 culture supernatant (medium: n = 4, WT and SK930, n = 3). **b** Growth curves of WT and SK930 strain (n = 3). 1×10^2 CFU ml^{-1} of each strain were added to the medium. After culturing for 24 hours, both bacterial numbers reached to 1×10^6 CFU ml^{-1}. **c** Measuring the amount of LPS (at $OD_{600} = 0.5$) of WT and SK930 strain (n = 3). The individual data were obtained from independent cultures. All data shown represent the mean ± SEM. Statistical significance was calculated using the one-way ANOVA followed by Tukey's post-hoc test (**a**) and the Welch's *t*-test (two-sided) (**c**).

Supplementary figure 3

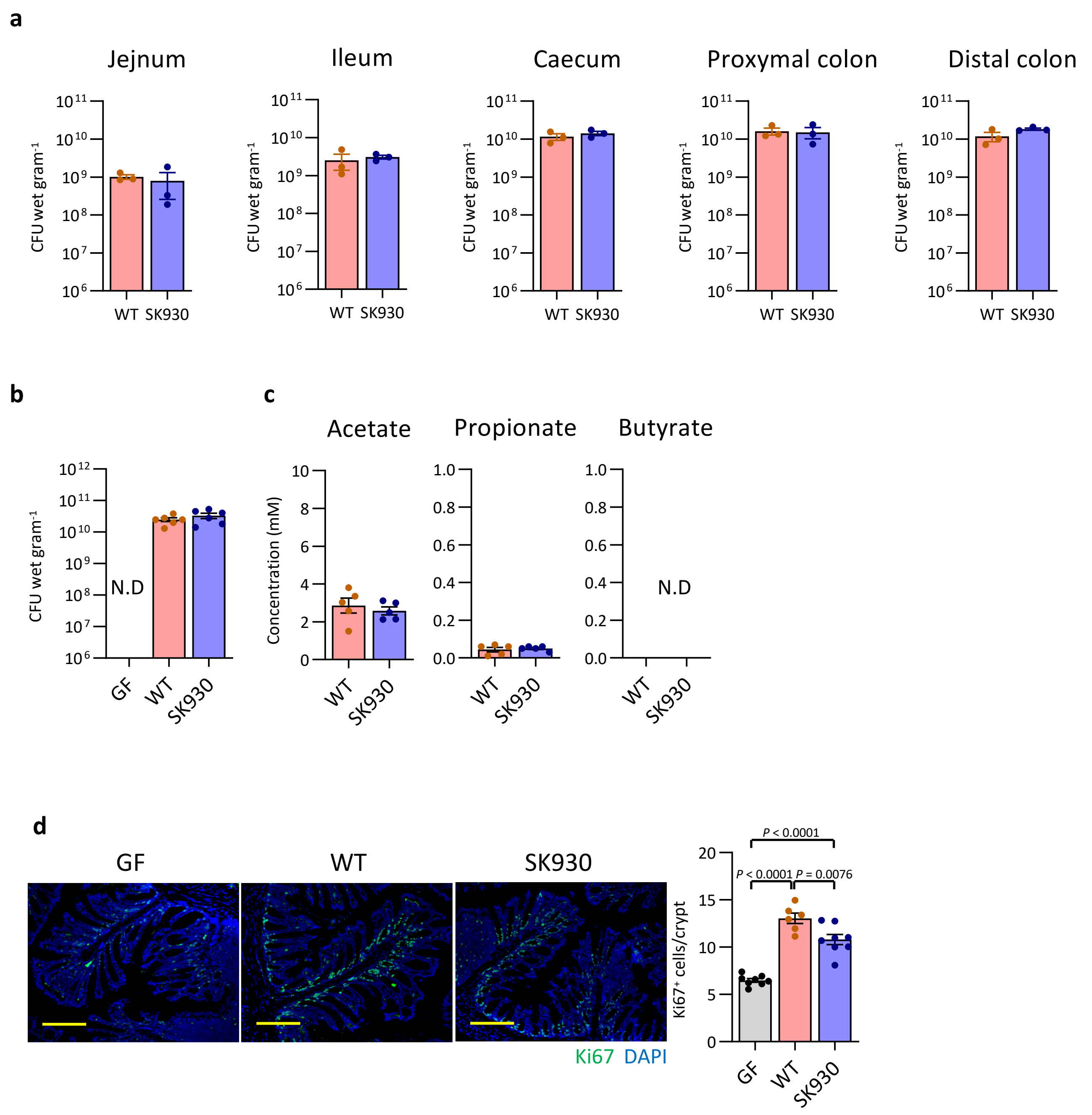

Supplementary Fig. 3. Characterisation of F1 gnotobiotic mice with WT or SK930 strain.
a The number of *E. coli* in the luminal contents from the jejunum to the distal colon obtained from the WT-strain-associated and SK930-strain-associated mice (n = 3, independent animals). **b** Faecal bacterial numbers of GF, WT or SK930 strain-associated mice (n = 6). **c** Faecal SCFA concentrations of WT and SK930 strain-associated mice (n = 5, independent animals). **d** Representative microscopic images of Ki67 immunostaining (green) in colonic tissue. Nuclei are counterstained with DAPI (blue). Scale bar: 100 µm (GF: n = 8, WT: n = 6, SK930: n = 8, independent animals). All data shown represent the mean ± SEM. Statistical significance was calculated using the Student's *t*-test (two-sided) (**a-c**), the one-way ANOVA followed by Tukey's post-hoc test (**d**). N.D. not detected.

Supplementary figure 4

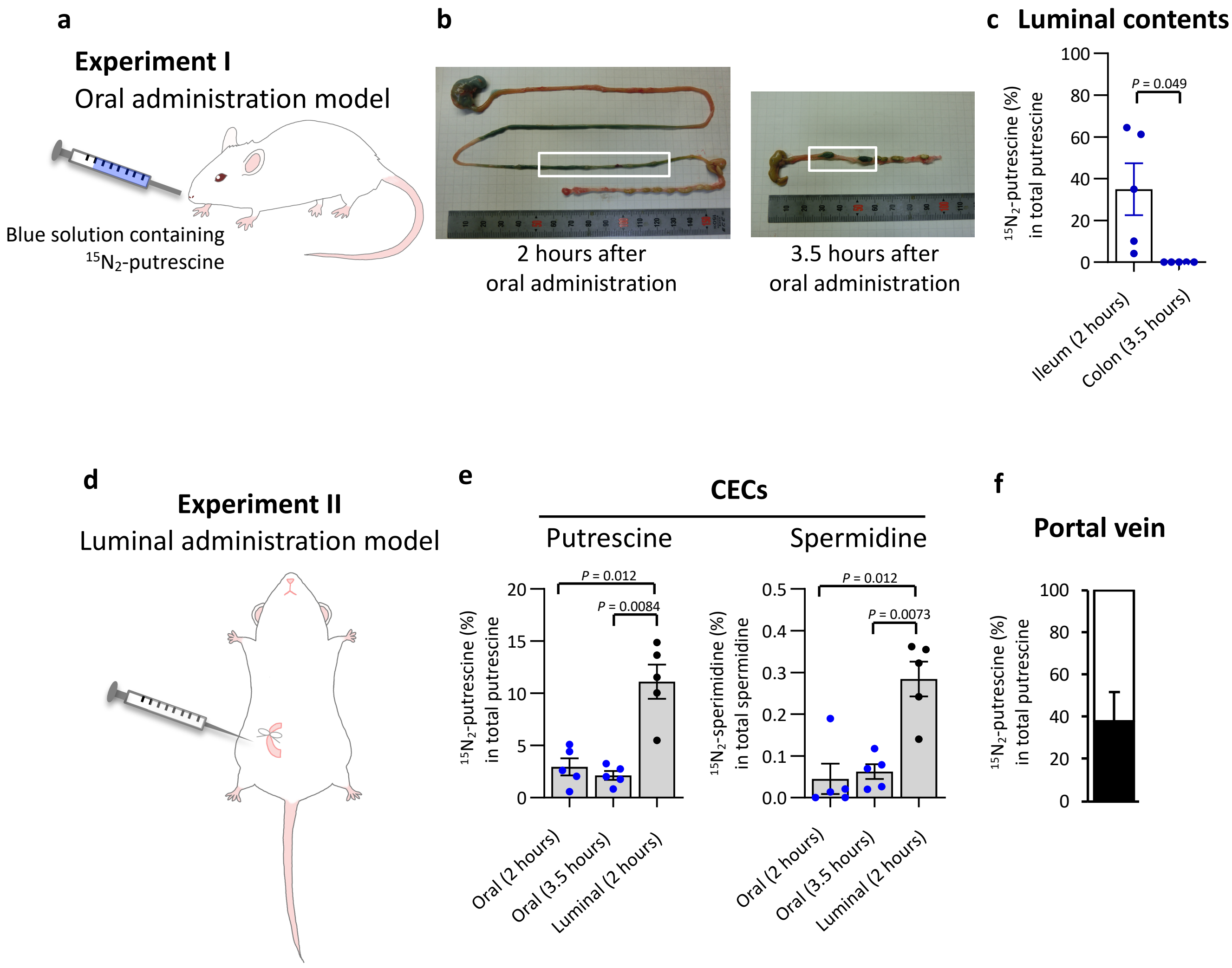

Supplementary Fig. 4. Comparison of uptake between dietary and colonic luminal polyamine into the host. Experiment I: **a** SPF mice were orally administered 200 μM stable-isotope labeled $^{15}N_2$-putrescine in D-PBS containing food coloring blue agent (200 μl). **b** White squares represent the intestinal regions analysed for the detection of polyamine. The luminal contents of these regions are blue. The lower part of the ileum 2 hours after oral administration (left panel, n = 5) and colon 3.5 hours after oral administration (right panel). **c** Isotope ratio of polyamines in the luminal contents (2 hours and 3.5 hours after oral administration.(n = 5, independent animals). **Experiment II**: **d** SPF mice were injected 200 μM isotope-labeled $^{15}N_2$-putrescine (200 μl) into the colonic lumen to assess the uptake of luminal putrescine into the CECs. Two hours post-injection, the isotope ratio of polyamines in the CECs was determined and compared with those in the CECs collected at 2 hours (the solution reached the lower small intestine) and 3.5 hours (the solution reached the colon) after oral administration in experiment I. **e** Isotope ratio of polyamines in CECs (n = 5, independent animals). **f** Proportion of stable-isotope labeled $^{15}N_2$-putrescine in portal vein (2 hours after luminal administration model) The black bar indicates the percent of $^{15}N_2$ putrescine enrichment in total putrescine (n = 5, independent animals). All data shown represent the mean ± SEM. Statistical significance was calculated using the Welch's *t*-test (two-sided) (**c**) and the Welch's ANOVA followed by Dunnett's T3 method (e).

Supplementary figure 5

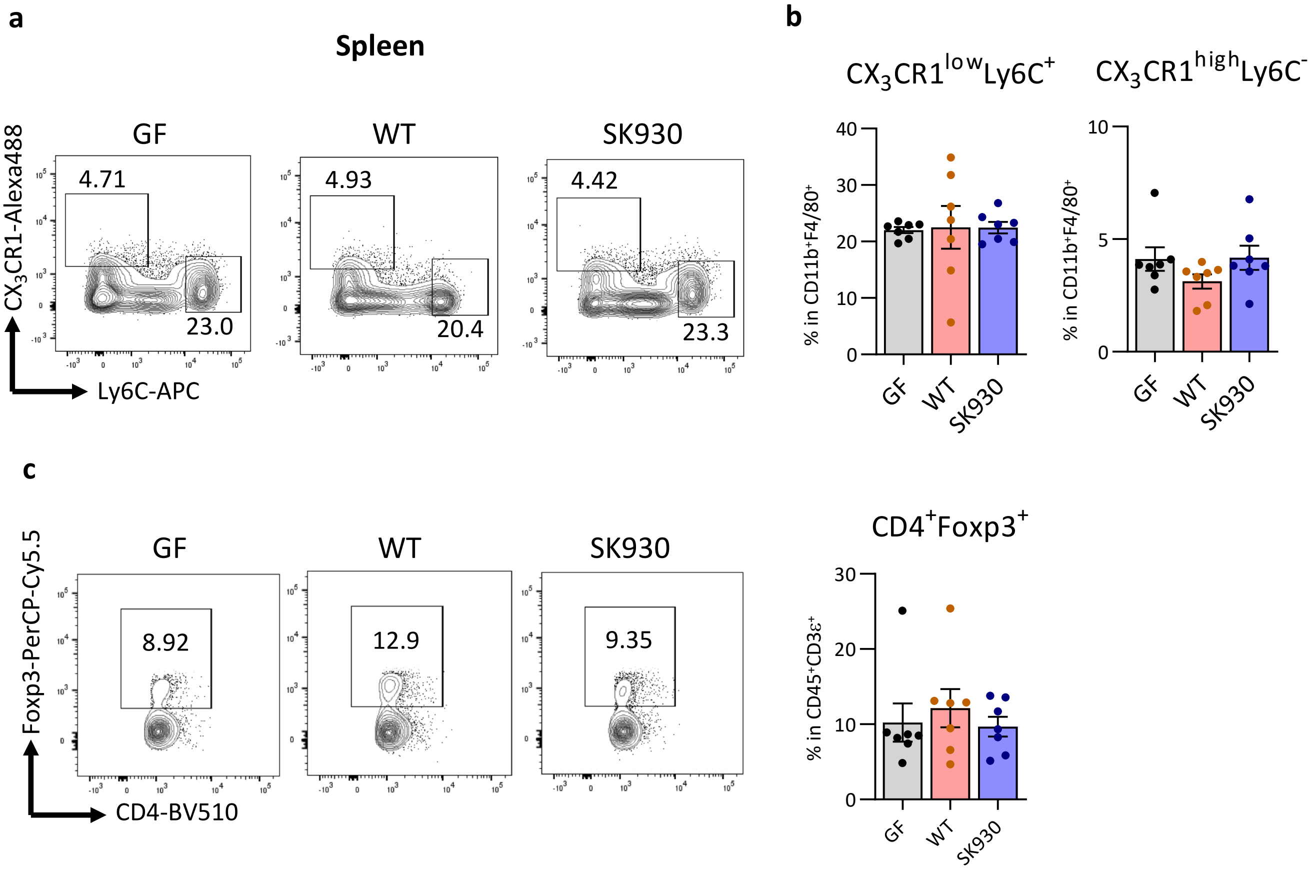

Supplementary Fig. 5. Splenic macrophage subsets and CD4⁺Foxp3⁺ cells in cLP of gnotobiotic mice under physiological conditions. a, **b** Representative flow cytometry images and frequency of $CX_3CR1^{high}Ly6C^{-}$ macrophage and $CX_3CR1^{low}Ly6C^{+}$ in live $CD45^{+}$ $CD11b^{+}$ $F4/80^{+}$ splenic cells of GF, WT or SK930 strain-associated mice (n = 7, independent animals). **c** Representative flow cytometry images and frequency of $CD4^{+}Foxp3^{+}$ cells in live $CD45^{+}$ $CD3\varepsilon^{+}$ cells in cLP of GF, WT- or SK930-strain-associated mice (n = 7, independent animals). Data shown represent the mean ± SEM. Statistical significance was calculated using the Welch's ANOVA followed by Dunnett's T3 post-hoc test (**b, c**).

Supplementary figure 6

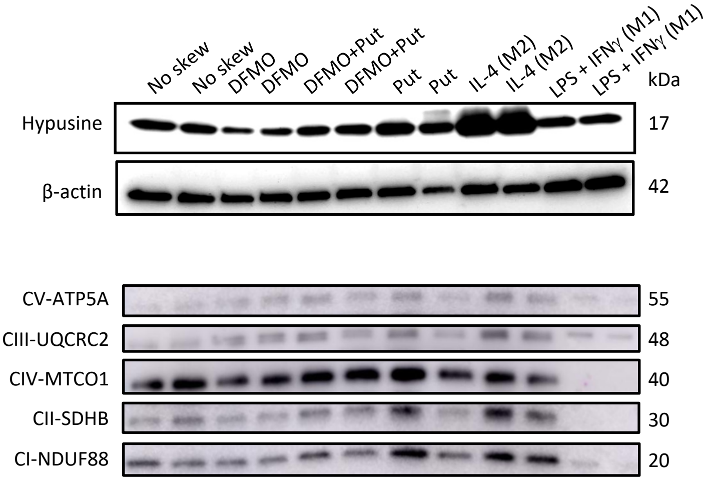

Supplementary Fig. 6. Effects of polyamines on the hyp-eIF5A level and respiration of BMDMs. BMDMs were cultured with macrophage colony-stimulating factor in the presence or absence of DFMO (1mM) and putrescine (100 μM) for 3 day. IL-4 was added for M2-skewing condition (20 ng/ml), and LPS and IFN-γ was added for M1-skewing condition (1 μg/ml and 1 ng/ml). The images were western blot analysis of hyp-eIF5A and OXPHOS complexes I-V. These data were obtained from one independent experiment.

Supplementary figure 7

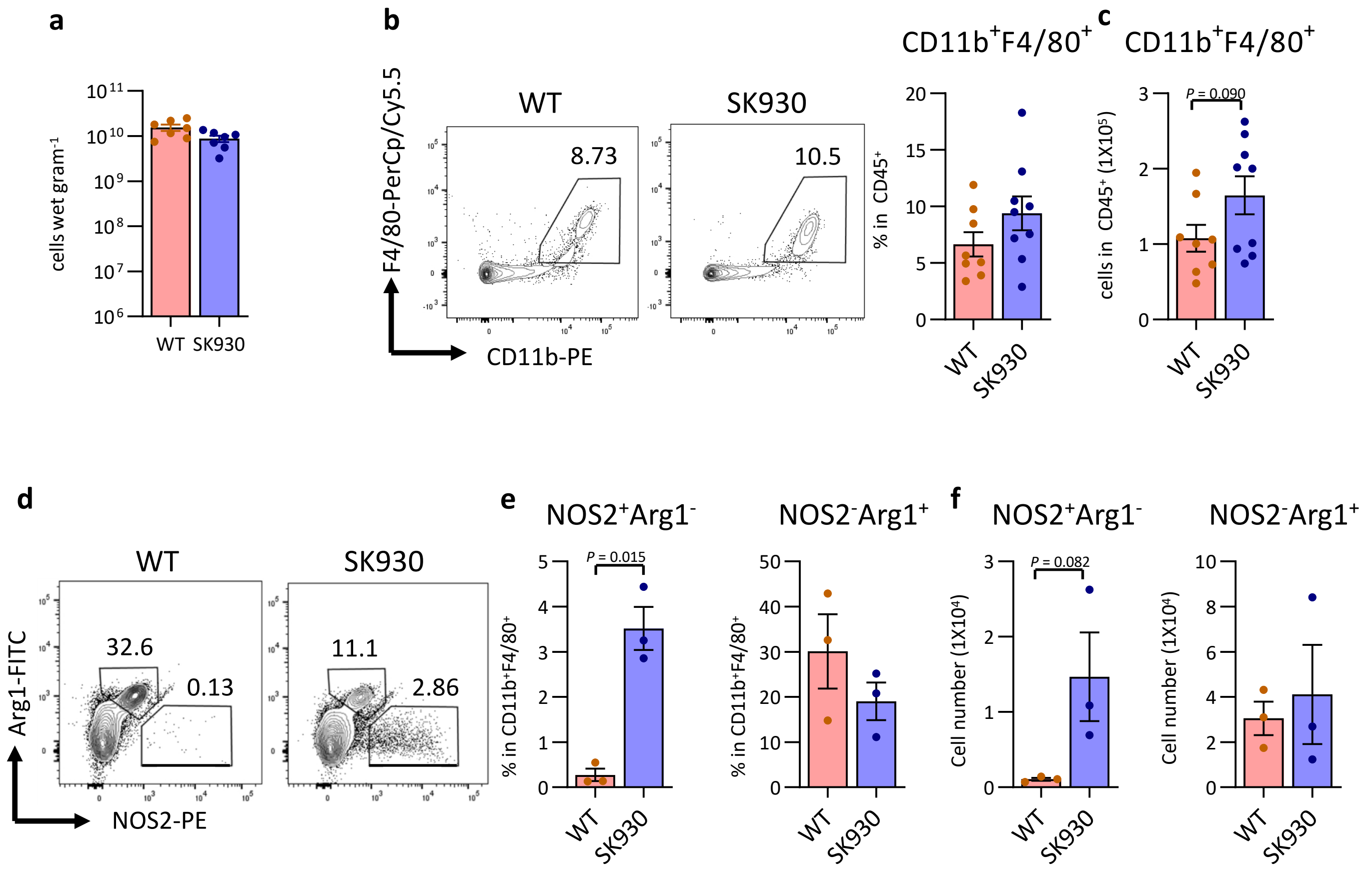

Supplementary Fig. 7. Flow cytometry analysis of macrophage in mice with DSS-induced colitis. a The number of *E. coli* in colonic contents of mice during DSS-induced colitis by quantitative PCR using *E. coli* specific primer (n = 7, independent animals) **b, c** Representative flow cytometry images, frequency and number of F4/80 positive cells in cLP of WT or SK930 strain-associated mice (WT: n = 8, SK930: n = 9, independent animals). **d–f** Representative flow cytometry images, frequency and numbers of $NOS2^{+}Arg1^{-}$ or $NOS2^{-}Arg1^{+}$ macrophage in $CD45^{+}CD11b^{+}F4/80^{+}$ population in cLP of WT or SK930 strain–associated mice (n = 3, independent animals). All data shown represent the mean ± SEM. Statistical significance was calculated using the Student's *t*-test (**c**, **f**) (two-sided) and the Welch's *t*-test (two-sided) (**e**).

Supplementary figure 8

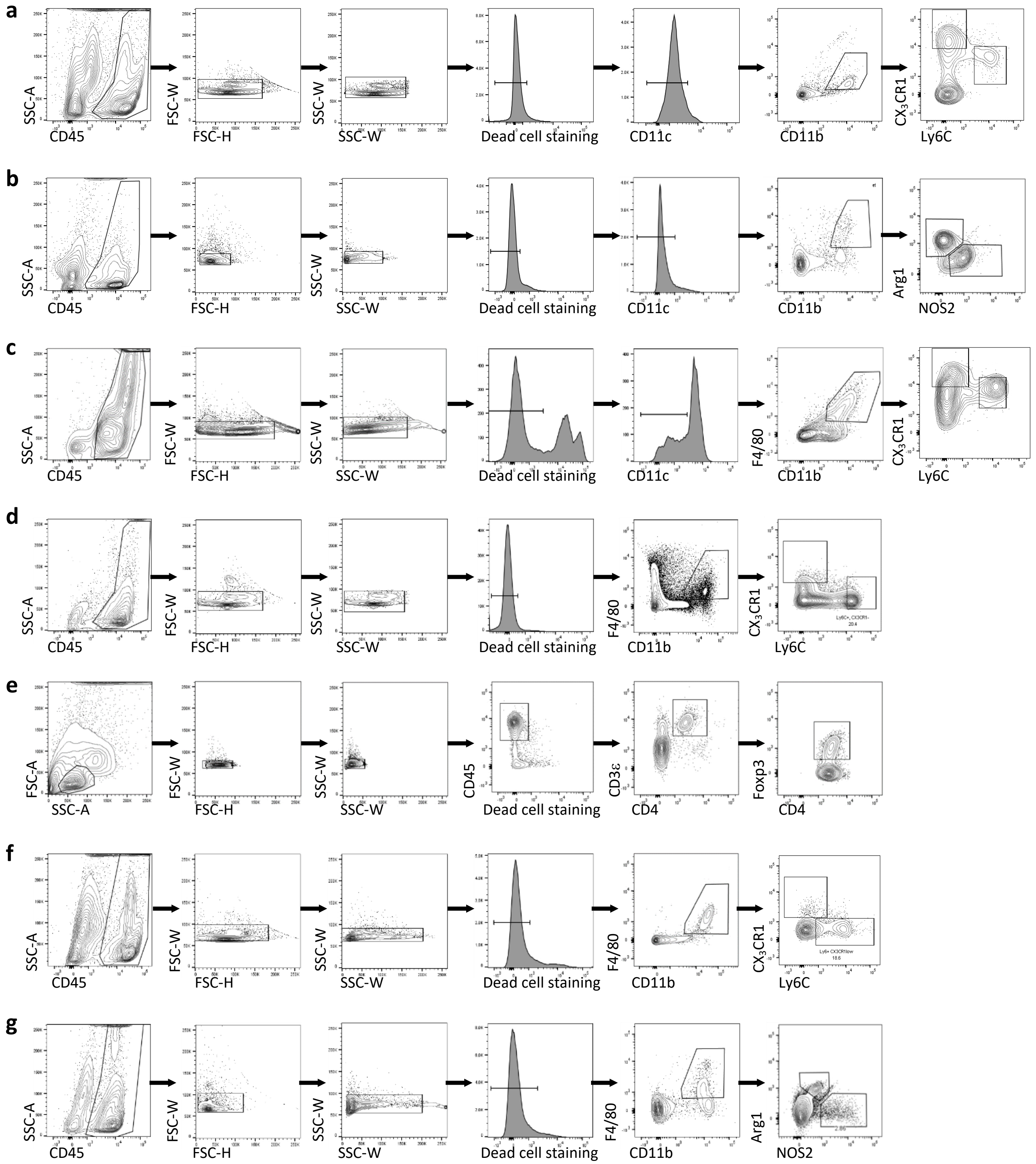

Supplementary Fig. 2. Gating strategies used for flow cytometry analysis.
a Gating strategy for $CX_3CR1^{high}Ly6C^{-}$ or $CX_3CR1^{low}Ly6C^{+}$ cells in $CD11c^{-}CD11b^{+}F4/80^{+}$ from cLP of mice presented on Fig. 3a. **b** Gating strategy for $NOS2^{-}Arg1^{+}$ cells or $NOS2^{+}Arg1^{-}$ in $CD11c^{-}CD11b^{+}F4/80^{+}$ cells from cLP of mice presented on Fig. 3d . **c** Gating strategy for $CX_3CR1^{high}Ly6C$ or $CX_3CR1^{low}Ly6C^{+}$ cells in $CD11c^{-}CD11b^{+}F4/80^{+}$ from BMDM cultures presented on Fig. 3h. **d** Gating strategy for $CX_3CR1^{high}Ly6C^{-}$ or $CX_3CR1^{low}Ly6C^{+}$ cells in $CD11b^{+}F4/80^{+}$ from spleen presented on Supplementary Fig. 5a. **e** $CD4^{+}Foxp3^{+}$ cells in $CD3ε^{+}$ from cLP of mice presented on Supplementary Fig. 5c. **f** Gating strategy for $CX_3CR1^{high}Ly6C^{-}$ or $CX_3CR1^{low}Ly6C^{+}$ cells in $CD11b^{+}F4/80^{+}$ from cLP of mice with DSS-induced colitis presented on Supplementary Fig. 5g and 7b. **g** Gating strategy for $NOS2^{-}Arg1^{+}$ cells or $NOS2^{+}Arg1^{-}$ in $CD11b^{+}F4/80^{+}$ cells from cLP of mice with DSS-induced colitis presented on Supplementary Fig. 7d.

Supplementary Table 1. Metabolome analysis of cecal contents in WT- and SK930-strain-associated mice

Metabolite	Concentration (μM)										WT / SK930	WT vs. SK930
	WT			SK			WT		SK			
	WT-1	WT-2	WT-3	SK-1	SK-2	SK-3	Mean	S.D.	Mean	S.D.	Ratio	*q* -value
**Putrescine**	139.87	175.27	170.82	9.83	6.05	7.89	161.99	19.28	7.92	1.89	20.44	0.13
Creatinine	514.84	411.69	596.75	219.06	120.70	150.37	507.76	92.74	163.38	50.45	3.11	0.13
S -Adenosylmethionine	18.44	19.98	20.31	11.61	8.17	12.42	19.58	0.99	10.73	2.25	1.82	0.13
Gly	1461.85	1010.23	1127.21	750.90	583.98	683.40	1199.76	234.39	672.76	83.97	1.78	0.37
3-Hydroxybutyric acid	143.28	328.26	259.19	98.80	54.43	N.D.	243.58	93.47	76.62	31.37	3.18	0.37
His	369.79	256.04	232.97	175.91	134.40	161.37	286.27	73.24	157.22	21.06	1.82	0.37
Lactic acid	1238.05	884.21	704.82	543.35	440.58	401.97	942.36	271.33	461.97	73.07	2.04	0.37
Choline	1572.20	2762.94	1900.30	1057.95	841.40	841.40	2078.48	615.04	913.58	125.02	2.28	0.37
Asn	70.66	42.16	37.17	32.06	18.16	17.04	50.00	18.07	22.42	8.37	2.23	0.37
Gluconic acid	68.96	106.96	188.51	222.65	240.47	168.19	121.48	61.08	210.44	37.65	0.58	0.37
Citrulline	17.66	9.46	13.57	8.99	6.55	7.43	13.56	4.10	7.66	1.24	1.77	0.37
Ser	237.56	155.53	126.56	105.34	85.24	85.32	173.22	57.58	91.97	11.58	1.88	0.37
Val	1409.66	953.79	763.81	733.02	499.01	546.99	1042.42	331.92	593.00	123.60	1.76	0.37
Ornithine	27.89	11.23	27.78	10.89	7.33	15.19	22.30	9.59	11.14	3.93	2.00	0.37
Thr	126.06	50.71	51.13	43.30	21.26	24.07	75.97	43.38	29.54	12.00	2.57	0.37
Betaine aldehyde_+H_2O	20.39	43.62	34.49	22.23	17.27	21.27	32.84	11.70	20.26	2.63	1.62	0.37
Ala	1805.12	895.29	739.60	736.46	410.71	474.24	1146.67	575.52	540.47	172.67	2.12	0.37
Ile	980.47	584.01	448.08	498.10	324.13	327.92	670.85	276.62	383.38	99.37	1.75	0.37
Tyr	640.93	290.98	258.75	231.52	136.45	167.77	396.89	211.96	178.58	48.45	2.22	0.37
Leu	1880.37	819.40	648.25	653.96	325.63	337.88	1116.01	667.47	439.16	186.13	2.54	0.37
Creatine	692.35	553.77	964.38	614.92	489.14	483.05	736.83	208.89	529.04	74.44	1.39	0.37
Phe	834.97	345.02	297.17	288.14	145.38	159.17	492.38	297.65	197.56	78.75	2.49	0.37
Citric acid	174.77	141.70	167.35	147.42	142.66	142.88	161.27	17.35	144.32	2.68	1.12	0.37
Lys	1464.27	654.17	735.99	648.26	426.57	523.43	951.47	445.97	532.75	111.13	1.79	0.37
Asp	114.11	38.70	53.01	48.98	23.51	29.25	68.61	40.05	33.91	13.36	2.02	0.37
Met	543.43	197.25	154.40	173.24	88.02	93.51	298.36	213.31	118.25	47.70	2.52	0.37
Arg	1573.97	509.46	806.70	612.58	372.21	515.20	963.37	549.28	500.00	120.90	1.93	0.37
Gln	675.92	169.54	170.76	184.07	29.31	67.30	338.74	292.00	93.56	80.65	3.62	0.37
Pro	1783.05	1601.69	2713.40	1443.37	1232.06	1981.78	2032.71	596.43	1552.41	386.57	1.31	0.39
Trp	37.47	9.51	9.16	10.50	4.04	5.46	18.71	16.24	6.67	3.40	2.81	0.39
Glu	2853.49	1615.94	2037.91	2082.92	1113.68	2005.29	2169.11	629.12	1733.96	538.58	1.25	0.47
Succinic acid	1360.37	1507.97	345.51	1107.22	132.18	736.74	1071.28	632.86	658.71	492.18	1.63	0.47
Cys	25.36	9.80	6.95	10.92	9.16	7.14	14.04	9.91	9.07	1.89	1.55	0.51
Uridine	39.14	42.43	49.23	41.42	43.63	54.00	43.60	5.15	46.35	6.71	0.94	0.62
GABA	11.93	9.19	20.95	13.67	10.97	15.14	14.02	6.15	13.26	2.11	1.06	0.86
Hydroxyproline	9.02	6.54	15.98	7.62	N.D.	6.06	10.51	4.89	-	-	-	-
Spermine	11.77	14.52	16.27	14.58	N.D.	17.35	14.19	2.27	-	-	-	-
Spermidine	55.79	45.38	47.63	8.22	N.D.	N.D.	49.60	5.48	-	-	-	-

N.D: not detected.

Supplementary Table 2. Primer sequence for quantitative PCR

Gene	Primer sequence	
18S rRNA	forward	5' - GGACCAGAGCGAAAGCATTTG - 3'
	reverse	5' - TTGCCAGTCGGCATCGTTTAT - 3'
Mki67	forward	5' - AATCCAACTCAAGTAAACGGGG - 3'
	reverse	5' - TTGGCTTGCTTCCATCCTCA - 3'
