# Supplementary material for: Symbiotic polyamine metabolism regulates epithelial proliferation and macrophage differentiation in the colon
Source: Nat Commun. 2021 Apr 8;12:2105. doi: 10.1038/s41467-021-22212-1 (PMC8032791; doi:10.1038/s41467-021-22212-1)
Supplement: Supplementary file 3 — Descriptions of Additional Supplementary Files [file 41467_2021_22212_MOESM3_ESM.docx]

Descriptions of Additional Supplementary Files

**Supplementary Data 1**

**Description:** Metabolome analysis of cecal contents in SPF and GF mice with or without fecal transplantation from SPF mice.

**Supplementary Data 2**

**Description:** Metabolome analysis of colonic epithelial cells in SPF and GF mice with or without fecal transplantation from SPF mice.
