# Supplementary material for: Symbiotic polyamine metabolism regulates epithelial proliferation and macrophage differentiation in the colon
Source: Nat Commun. 2021 Apr 8;12:2105. doi: 10.1038/s41467-021-22212-1 (PMC8032791; doi:10.1038/s41467-021-22212-1)
Supplement: Supplementary file 6 — Reporting Summary [file 41467_2021_22212_MOESM6_ESM.pdf]

nature research

Corresponding author(s): Koji Hase

Last updated by author(s): Feb 14, 2021

Reporting Summary

Nature Research wishes to improve the reproducibility of the work that we publish. This form provides structure for consistency and transparency in reporting. For further information on Nature Research policies, see our Editorial Policies and the Editorial Policy Checklist.

Statistics

For all statistical analyses, confirm that the following items are present in the figure legend, table legend, main text, or Methods section.

n/a	Confirmed	
☐	☒	The exact sample size (*n*) for each experimental group/condition, given as a discrete number and unit of measurement
☐	☒	A statement on whether measurements were taken from distinct samples or whether the same sample was measured repeatedly
☐	☒	The statistical test(s) used AND whether they are one- or two-sided<br>*Only common tests should be described solely by name; describe more complex techniques in the Methods section.*
☒	☐	A description of all covariates tested
☐	☒	A description of any assumptions or corrections, such as tests of normality and adjustment for multiple comparisons
☐	☒	A full description of the statistical parameters including central tendency (e.g. means) or other basic estimates (e.g. regression coefficient) AND variation (e.g. standard deviation) or associated estimates of uncertainty (e.g. confidence intervals)
☐	☒	For null hypothesis testing, the test statistic (e.g. *F*, *t*, *r*) with confidence intervals, effect sizes, degrees of freedom and *P* value noted<br>*Give P values as exact values whenever suitable.*
☒	☐	For Bayesian analysis, information on the choice of priors and Markov chain Monte Carlo settings
☒	☐	For hierarchical and complex designs, identification of the appropriate level for tests and full reporting of outcomes
☒	☐	Estimates of effect sizes (e.g. Cohen's *d*, Pearson's *r*), indicating how they were calculated

Our web collection on statistics for biologists contains articles on many of the points above.

Software and code

Policy information about availability of computer code

Data collection	I. To measure polyamine concentrations, we used gas chromatography-mass spectrometry (GC-MS; GCMS-QP2010, Shimadzu, Co., Japan)<br>II. To collecting microscopic data, we used confocal fluorescence microscope LSM 880 (ZEISS, Germany) for LC3 and p62 immunostaining and BZ-9000 fluorescence microscope (KEYENCE, Japan) for Ki-67 immunostaining, EdU assay and HE staining.<br>III. For flow cytometry analysis, we used LSR II flow cytometer (BD Biosciences) with FACSDiva™ software Ver. 8.0 (BD Biosciences).<br>IV. For metabolome analysis, we used the Agilent CE System, the Agilent G3250AA LC/MSD TOF System, the Agilent 1100 Series Binary HPLC Pump, the G1603A Agilent CE-MS adapter and the G1607A Agilent CE-ESI-MS Sprayer Kit (Agilent Technologies, USA).
Data analysis	I. GC-MS data were analyzed using GCMSSolution (ver.2, Shimadzu, Co., Japan)<br>II. All microscopic data were analyzed using ImageJ software (ver. 1.47, National Institutes of Health, USA).<br>III. Flow cytometry data were analyzed using FlowJo (ver. 10.6, FlowJo, USA).<br>IV. For CE-TOFMS data processing, quantification and peak annotation, in-house software (MasterHands) was used (Metabolomics 6, 78-95, 2010). Heatmaps were drawn with normalised z-score using R software (ver. 3.5.3).<br>V. Prism GraphPad Software ver. 8.4 was used for statistical analysis.

For manuscripts utilizing custom algorithms or software that are central to the research but not yet described in published literature, software must be made available to editors and reviewers. We strongly encourage code deposition in a community repository (e.g. GitHub). See the Nature Research guidelines for submitting code & software for further information.

Data

Policy information about availability of data

All manuscripts must include a data availability statement. This statement should provide the following information, where applicable:

- Accession codes, unique identifiers, or web links for publicly available datasets
- A list of figures that have associated raw data
- A description of any restrictions on data availability

The dataset generated and analyzed during the current study are available from the corresponding authors upon reasonable request.

Field-specific reporting

Please select the one below that is the best fit for your research. If you are not sure, read the appropriate sections before making your selection.

[x] Life sciences   [ ] Behavioural & social sciences   [ ] Ecological, evolutionary & environmental sciences

For a reference copy of the document with all sections, see nature.com/documents/nr-reporting-summary-flat.pdf

Life sciences study design

All studies must disclose on these points even when the disclosure is negative.

Sample size	The sample size was empirically started at 3 -5, and was increased at the next experiment when there was no significant difference. For gnotobiotic mice, the experiment was conducted using the maximum number of individuals that could be used.
Data exclusions	No data was excluded in all our experiments.
Replication	Fig 1a, d, e, f, Fig 2d, g, h, Fig 4a, b and Supplementary Fig. 3d were performed two independent experiments. These attempts obtained similar results.<br>Fig 5a-d were performed three independent experiments. The experiment was carried out 3 times and the reproducibility was confirmed in 2 times.Other experiminets were performed one independent experiments.
Randomization	All samples in this study were randomly allocated.
Blinding	In histological scoring of Fig. 5e , the investigator was blind to the experimental groups during the analyses and data collection.<br>In other experiments except histological scoring, we were not blinded to group allocation during data collection and analysis. Because the data collection and analysis were performed with quantitative instruments to maintaining objectivity.

Reporting for specific materials, systems and methods

We require information from authors about some types of materials, experimental systems and methods used in many studies. Here, indicate whether each material, system or method listed is relevant to your study. If you are not sure if a list item applies to your research, read the appropriate section before selecting a response.

Materials & experimental systems

n/a	Involved in the study	
[ ]	[x]	Antibodies
[x]	[ ]	Eukaryotic cell lines
[x]	[ ]	Palaeontology and archaeology
[ ]	[x]	Animals and other organisms
[x]	[ ]	Human research participants
[x]	[ ]	Clinical data
[x]	[ ]	Dual use research of concern

Methods

n/a	Involved in the study	
[x]	[ ]	ChIP-seq
[ ]	[x]	Flow cytometry
[x]	[ ]	MRI-based neuroimaging

Antibodies

Antibodies used

For immunostaining:
Antibody|Conjugation| Supplier| Catalogue number| Clone| Dilution
rabbit anti-Ki67|non|Novocastra|NCL-Ki67p|polyclonal|1:1000
rabbit anti-LC3|non|Medical and Biological Laboratories|PM036|polyclonal|1:1000
guinea pig anti-p62|non|Medical and Biological Laboratories|PM066|polyclonal|1:200
goat anti-rabbit-IgG|Alexa Fluor 488|Thermo Fisher Scientific|A-11034|polyclonal|1:400
goat anti-guinea pig-IgG|biotin|Vector Laboratories|BA-7000|polyclonal|1:200

goat anti-rabbit-IgG|Alexa Fluor 488|Thermo Fisher Scientific|A-11008|polyclonal|1:500

For western blotting:
Antibody|Conjugation|Supplier| Catalogue number| Clone| Dilution
rabbit anti-hypusine antibody|Merck Millipore|ABS1064|1:5000
mouse anti-β-actin antibody|Sigma-Aldrich|A1978|AC-15|1:2500
mouse total OXPHOS antibody|Abcam|ab110413|monoclonal cocktail: 20E9DH10C12, 21A11AE7, 1D6E1A8 15H4C4|1:1000
mouse anti-actin antibody|Wako|017-2455, Wako, Japan)|6D1|1:5000
donkey anti-rabbit IgG|HRP|Cytiva|NA934|polyclonal|1:10000
sheep anti-mouse IgG|HRP|Cytiva|NA931|polyclonal|1:10000
horse anti-mouse IgG|HRP|CST|7076|polyclonal|1:2000

For flow cytometry:
Antibody|Conjugation|Supplier| Catalogue number| Clone| Dilution
rat anti-mouse CD16/CD32|BioLegend|101302|AT-10|1:400
rat anti-mouse CD45|BV 510|BioLegend|103138|30-F11|1:400
rat anti-mouse CD11b|FITC|Thermo Fisher Scientific|11-0112-85|M1/70|1:200
rat anti-mouse CD11b|PE|Thermo Fisher Scientific|12-0112-83|M1/70|1:200
rat anti-mouse CD11b|BUV737|BD Biosciences|564443|M1/70|1:200
hamster anti-mouse CD11c|BUV395|BD Biosciences|564080|HL3|1:400
rat anti-mouse F4/80|PerCP/Cy5.5|BioLegend|123127|BM8|1:200
rat anti-mouse F4/80|APC|BioLegend|123115|BM8|1:200
mouse anti-mouse CX3CR1|PE|BioLegend|149005|SA011F11|1:200
goat anti-mouse CX3CR1|Alexa Fluor 488|R&D Systems|FAB5825G|polyclonal|1:50
rat anti-mouse Ly6C|APC|BD Biosciences|560595|AL-21|1:200
rat anti-mouse NOS2|PE|Thermo Fisher Scientific|12-5920-80|CXNFT|1:100
sheep anti-mouse/human Arg1|FITC|R&D Systems|IC5868F|1:100
rat anti-mouse CD45|BUV395|BD Biosciences|564279|30-F11|1:400
hamster anti-mouse CD3e|BUV737|BD Biosciences|564618|145-2C11|1:200
rat anti-mouse CD4|BV510|BioLegend|100559|RM4-5|1:200
rat anti-mouse Foxp3|PerCP-Cy5.5|Thermo Fisher Scientific|45-5773-82|FJK-16s1:200

Validation

For immunestaining:
anti-Ki67|NCL-Ki67p|Confirmed in the manufacturer's data sheet|
anti-LC3|PM036|https://ruo.mbl.co.jp/bio/dtl/A/?pcd=PM066
anti-p62|PM066|https://ruo.mbl.co.jp/bio/dtl/A/?pcd=PM066
anti-rabbit-IgG|https://www.thermofisher.com/antibody/product/Goat-anti-Rabbit-IgG-H-L-Highly-Cross-Adsorbed-Secondary-Antibody-Polyclonal/A-11034
anti-guinea pig-IgG|BA-7000|Confirmed in the manufacturer's data sheet|
anti-rabbit-IgG|A-11008|https://www.thermofisher.com/antibody/product/Goat-anti-Rabbit-IgG-H-L-Highly-Cross-Adsorbed-Secondary-Antibody-Polyclonal/A-11008

For western blotting:
anti-hypusine|ABS1064|https://www.merckmillipore.com/JP/ja/product/Anti-Hypusine,MM_NF-ABS1064-I-100UL
anti-B-actin|AC-15|https://www.sigmaaldrich.com/catalog/product/sigma/a1978?lang=ja&region=JP&cm_sp=Insite-_-caSrpResults_srpRecs_srpModel_a1978-_-srpRecs3-1
total OXPHOS antibody|https://www.abcam.co.jp/total-oxphos-rodent-wb-antibody-cocktail-ab110413.html
anti-actin antibody|017-2455|Confirmed in the manufacturer's call center|
anti-rabbit IgG|NA934|https://cdn.cytivalifesciences.com/dmm3bwsv3/AssetStream.aspx?mediaformatid=10061&destinationid=10016&assetid=13497
anti-mouse IgG|NA931|https://cdn.cytivalifesciences.com/dmm3bwsv3/AssetStream.aspx?mediaformatid=10061&destinationid=10016&assetid=13495
anti-mouse IgG|7076|https://www.cellsignal.jp/products/secondary-antibodies/anti-mouse-igg-hrp-linked-antibody/7076|

For flow cytometry:
anti-mouse CD16/CD32|https://www.biolegend.com/ja-jp/products/purified-anti-mouse-cd16-32-antibody-190
anti-mouse CD45|103138|https://www.biolegend.com/ja-jp/products/brilliant-violet-510-anti-mouse-cd45-antibody-7995
anti-mouse CD11b|11-0112-85|https://www.thermofisher.com/antibody/product/CD11b-Monoclonal-Antibody-M1-70-FITC-eBioscience/11-0112-85
anti-mouse CD11b|12-0112-83|https://www.thermofisher.com/antibody/product/CD11b-Monoclonal-Antibody-M1-70-FITC-eBioscience/12-0112-83
anti-mouse CD11b|564443|https://www.bdbiosciences.com/ds/pm/tds/564443.pdf
anti-mouse CD11c|564080|https://www.bdbiosciences.com/ds/pm/tds/564080.pdf
anti-mouse F4/80 |123127|https://www.biolegend.com/ja-jp/products/percp-cyanine5-5-anti-mouse-f480-antibody-4303
anti-mouse F4/80|123115|https://www.biolegend.com/ja-jp/products/apc-anti-mouse-f4-80-antibody-4071?GroupID=GROUP20
anti-mouse CX3CR1|149005|https://www.biolegend.com/ja-jp/products/pe-anti-mouse-cx3cr1-antibody-10376?GroupID=GROUP20
anti-mouse CX3CR1|FAB5825G|https://www.rndsystems.com/products/mouse-cx3cr1-alexa-fluor-488-conjugated-antibody_fab5825g
anti-mouse Ly6C|560595|https://www.bdbiosciences.com/ds/pm/tds/560595.pdf

anti-mouse NOS2|12-5920-80|https://www.thermofisher.com/antibody/product/iNOS-Antibody-clone-CXNFT-Monoclonal/12-5920-82
anti-mouse/human Arg1|IC5868F|https://www.rndsystems.com/products/human-mouse-arginase-1-arg1-fluorescein-conjugated-antibody_ic5868f
anti-mouse CD45|564279|https://www.bdbiosciences.com/ds/pm/tds/564279.pdf
anti-mouse CD3e|564618|https://www.bdbiosciences.com/ds/pm/tds/564618.pdf
anti-mouse CD4|100559|https://www.biolegend.com/ja-jp/products/brilliant-violet-510-anti-mouse-cd4-antibody-7991
anti-mouse FoxP3|45-5773-82|https://www.thermofisher.com/antibody/product/FOXP3-Antibody-clone-FJK-16s-Monoclonal/14-5773-82

Animals and other organisms

Policy information about studies involving animals; ARRIVE guidelines recommended for reporting animal research

Laboratory animals	GF (IQI/Jic), C57BL6/J and ICR/jcl mice were purchased from CLEA Japan, Inc. (Tokyo, Japan). GF (IQI/Jic) mice were bred at Kyodo Milk Industry Co., Ltd.<br>IQI/Jic mice (male; 8-12 week old) were used gnotobiotic experiments except (Fig. 5a-c, 5d-j).In the DSS experiment (Fig. 5a-c, 5d-j), mice were used without discrimination between males and females.<br>C57BL/6J mice (male; 6-12 week old) were used for colonic organoids and BMDM experiments.<br>ICR/jcle (male; 7 week old) were used for GC7 administration experiments(Fig. 5j, k) and for putrescine trace experiments (Supplementary Fig. 2).
Wild animals	This study did not involve wild animals.
Field-collected samples	This study did not involve samples collected from the field.
Ethics oversight	All animal experiments were approved by the 'Kyodo Milk Industry Co., Ltd. Animal Care and Use Committee' and were in accordance with the Guide for the Care and Use of Laboratory Animals, published by the National Academies Press.

Note that full information on the approval of the study protocol must also be provided in the manuscript.

Flow Cytometry

Plots

Confirm that:

- [x] The axis labels state the marker and fluorochrome used (e.g. CD4-FITC).
- [x] The axis scales are clearly visible. Include numbers along axes only for bottom left plot of group (a 'group' is an analysis of identical markers).
- [x] All plots are contour plots with outliers or pseudocolor plots.
- [x] A numerical value for number of cells or percentage (with statistics) is provided.

Methodology

Sample preparation	Colonic tissues were treated with HBSS containing 1 mM dithiothreitol, 20 mM ethylenediaminetetraacetic acid (EDTA) and 12.5 mM HEPES at 37°C for 30 min on a stirrer. The tissues were then minced and dissociated in RPMI-1640 containing 0.5 mg/ml collagenase (Wako), 0.125 mg/ml DNase I, 2% new-born calf serum, 100 U/ml penicillin, 100 µg/ml streptomycin and 20 mM HEPES (2R media) at 37°C for 30 min to obtain single-cell suspensions.<br>Single splenic leukocyte suspensions were prepared by mechanically disrupting the tissues through 100-µm nylon-mesh cell strainers (BD Biosciences) in 2R media. Red blood cells were removed by RBC lysis reagent.<br>These preparations were then subjected to flow cytometry analysis using the LSR II flow cytometer (BD Biosciences).
Instrument	LSR II (BD Biosciences)
Software	Data analysis was performed using FlowJo (ver. 10.6, FlowJo)
Cell population abundance	In this study, cell sorting was not performed.
Gating strategy	For detection of lymphoid cell populations, macrophage and lymphocyte were gated on a forward scatter (FSC)/side scatter (SSC) plot. Dead cells were excluded as Fixable Viability Stain (FVS) 780 positive cells (BD Biosciences).<br>Detailed gating strategies were shown as below.<br><br>Figure 4<br>initial gating: FVS780-negative CD45-BV510-positive →<br>CD11c-BUV395-negative →<br>CD11b-positive F4/80 positive →

a, h: CX3CR1-PE-high Ly6C-APC-positive or CX3CR1-PE-low Ly6C-APC-negative
d: Arg1-FITC-positive NOS2-PE-negative or Arg1-FITC-negative NOS2-PE-positive

Figure 5
initial gatings: FVS780-negative CD45-BV510-positive →
CD11b-positive F4/80 positive →
g: CX3CR1-PE-high Ly6C-APC-positive or CX3CR1-PE-low Ly6C-APC-negative

Supplementary Figure 5
initial gatings: FVS780-negative CD45-BV510-positive →
CD11b-positive F4/80 positive →
a: CX3CR1-PE-high Ly6C-APC-positive or CX3CR1-PE-low Ly6C-APC-negative

Supplementary Figure 5
initial gatings: FVS780-negative CD45-BUV395-positive →
CD3e-BUV737-positive CD4-BV510-positive →
c: CD4-BV510-positive FoxP3-PerCP-Cy5.5-positive

Supplementary Figure 7
initial gatings: FVS780-negative CD45-BV510-positive →
b: CD11b-positive F4/80 positive →
d: Arg1-FITC-positive NOS2-PE-negative or Arg1-FITC-negative NOS2-PE-positive

☒ Tick this box to confirm that a figure exemplifying the gating strategy is provided in the Supplementary Information.
